# Quercetin as a therapeutic agent for acute pancreatitis: a comprehensive review of antioxidant, anti-inflammatory, and immunomodulatory mechanisms

**DOI:** 10.3389/fphar.2025.1587314

**Published:** 2025-04-28

**Authors:** Zeyi Jiang, Gamar Lhamo, Mengjie Ma, Xuxia Ye, Jin Chen, Yibo He, Jian Xu, Liquan Huang

**Affiliations:** ^1^ The First Affiliated Hospital of Zhejiang Chinese Medical University (Zhejiang Provincial Hospital of Chinese Medicine), Hangzhou, Zhejiang, China; ^2^ School of Medical Technology and Information Engineering, Zhejiang Chinese Medical University, Hangzhou, Zhejiang, China; ^3^ Hospital of Azha Town, Naqu, Xizang, China

**Keywords:** quercetin, acute pancreatitis, antioxidant, anti-inflammatory, immunomodulatory, bioavailability

## Abstract

Acute pancreatitis (AP) is a severe inflammatory disorder of the pancreas, characterized by high morbidity and mortality rates. Despite significant advancements in understanding the pathophysiological mechanisms of AP, current treatment options still face considerable limitations. Recent studies have underscored the therapeutic potential of quercetin, a natural flavonoid, due to its potent antioxidant, anti-inflammatory, and immunomodulatory properties, positioning it as a promising therapeutic candidate for AP. This review explores the effects of quercetin on AP, highlighting its antioxidant activities, its role in immune modulation, and its protective effects on pancreatic tissue. Furthermore, it examines quercetin’s multi-target mechanisms and its advantages over conventional therapies, such as N-acetylcysteine and corticosteroids. Although preliminary studies suggest that quercetin can alleviate inflammation and oxidative stress in AP, clinical evidence remains limited. One of the main challenges for quercetin’s clinical application is its low bioavailability. Future research should focus on strategies to enhance its bioavailability and on conducting large-scale randomized controlled trials to more comprehensively assess its efficacy and safety in the treatment of AP.

## 1 Introduction

**FIGURE 1:**
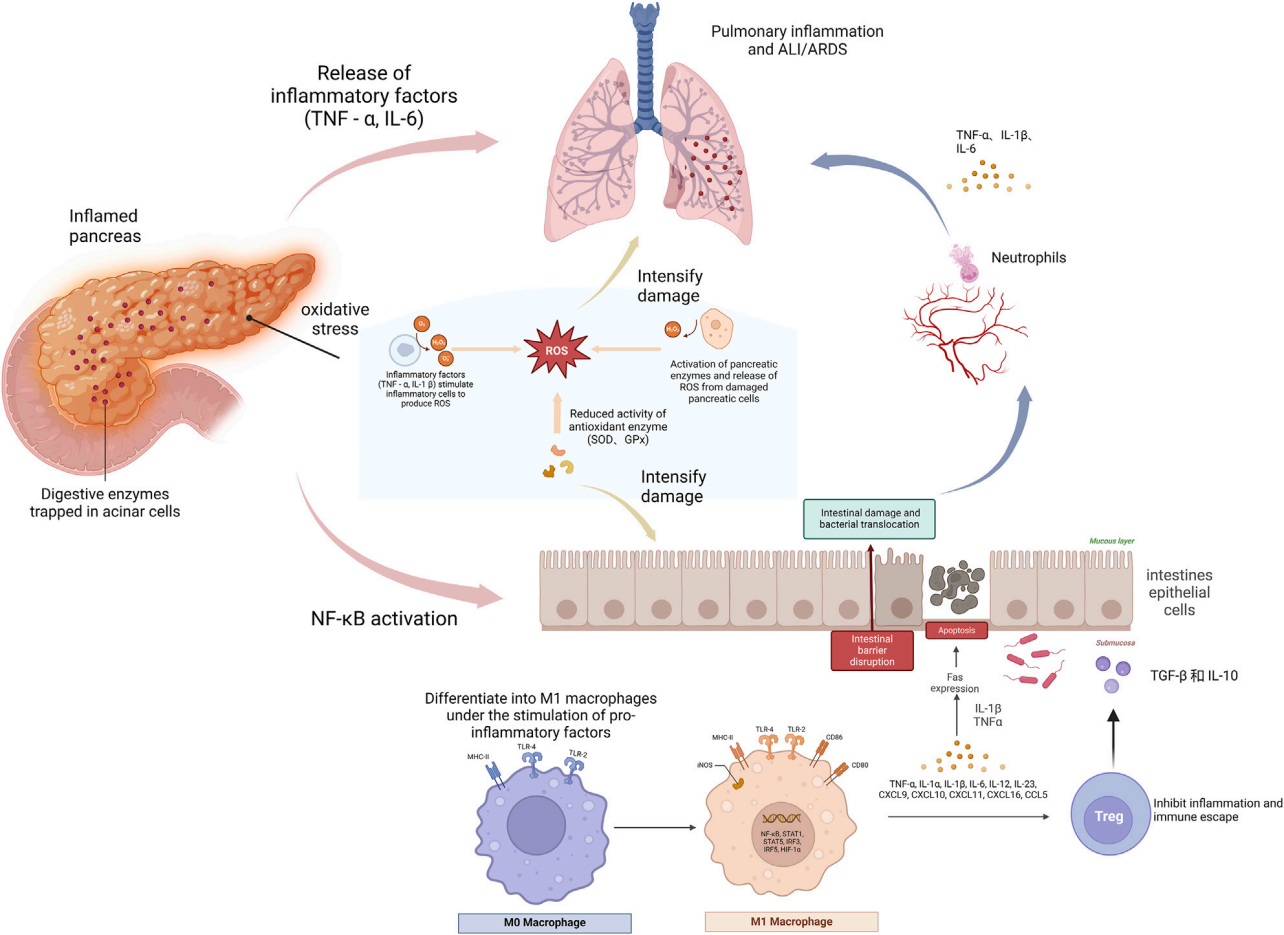
The role of pancreatic intestinal lung pathway in acute pancreatitis. This figure illustrates the progression of acute pancreatitis (AP), beginning with localized inflammation in the pancreas. Digestive enzymes become trapped in acinar cells, leading to oxidative stress, which subsequently triggers the release of inflammatory molecules such as tumor necrosis factor-alpha (TNF-α) and interleukin-6 (IL-6), exacerbating tissue damage. As inflammation spreads, bacteria and endotoxins are transported from the pancreas into the bloodstream and lungs, further amplifying the damage and potentially leading to complications such as acute lung injury (ALI) or acute respiratory distress syndrome (ARDS). Moreover, inflammatory mediators stimulate macrophages to differentiate into the pro-inflammatory M1 phenotype, which exacerbates the inflammatory response. The figure underscores the interconnectedness of the pancreas, intestines, and lungs in the pathogenesis of AP, illustrating how the systemic inflammatory response unfolds.

AP is a severe inflammatory condition of the pancreas, characterized by the rapid onset of inflammation, primarily driven by the premature activation of digestive enzymes within the pancreas. This condition can vary from mild, self-limiting episodes to severe forms that may progress to systemic inflammatory response syndrome (SIRS) and multiple organ dysfunction syndrome (MODS), ultimately leading to high morbidity and mortality rates (Windsor and McClave, 2023; Zhang Haoyu et al., 2024). AP can lead to severe systemic complications, including acute lung injury (ALI) and intestinal damage, both of which are life-threatening. These complications arise from inflammatory crosstalk between the pancreas, lungs, and intestines (Zhang Zhihang et al., 2024; Swetha et al., 2025; Shen et al., 2025). Figure 1 illustrates this intricate interaction, known as the ‘pancreas-intestine-lung’ pathway, which encompasses intestinal barrier disruption, bacterial translocation, and the widespread activation of inflammatory cascades. This pathway visually demonstrates how inflammation in the pancreas can trigger systemic complications and affect multiple organs. The pathophysiology of AP is strongly influenced by oxidative stress, which results from an imbalance between free radicals and antioxidant defenses, leading to cellular damage. These mechanisms drive the progression from localized pancreatic injury to widespread systemic inflammation and MODS (Zhang Chuan et al., 2024; Li et al., 2021). Although therapeutic strategies such as fluid resuscitation, nutritional support, and pain management are available, their effectiveness remains limited, and no specific pharmacological intervention exists that can halt or reverse disease progression (Tenner et al., 2024)^,^.

Quercetin, a naturally occurring flavonoid found in various fruits and vegetables, has attracted considerable attention as a potential therapeutic agent for AP due to its potent antioxidant, anti-inflammatory, and immunomodulatory properties (Li Ke et al., 2024). Structurally, quercetin belongs to the flavonol class of flavonoids, distinguished by hydroxyl groups (-OH) attached to the aromatic rings. These hydroxyl groups are crucial to quercetin’s ability to scavenge free radicals and chelate metal ions, both of which are key mechanisms in mitigating oxidative stress (Ungurianu et al., 2024; Li X. et al., 2024). Through these actions, quercetin neutralizes reactive oxygen species (ROS) and chelates metal ions (Kashyap et al., 2019; Chiang et al., 2023), which significantly reduce oxidative damage, a key pathological factor in AP. By scavenging ROS, quercetin prevents cellular damage and tissue inflammation, thereby helping to attenuate disease progression. In addition to its antioxidant effects, quercetin also exhibits significant anti-inflammatory properties (Beken et al., 2020; Goyal and Agrawal, 2022). It achieves this by inhibiting key inflammatory mediators such as nuclear factor-kappa B (NF-κB), tumor necrosis factor-alpha (TNF-α), and interleukin-1 beta (IL-1β), all of which play central roles in the inflammatory pathways driving the pathogenesis of AP (Wang et al., 2023; Nagy-Pénzes et al., 2022). Beyond its anti-inflammatory effects, quercetin also regulates immune cell functions. It inhibits NF-κB, reducing the expression of pro-inflammatory cytokines, while modulating TNF-α and IL-1β to help reduce systemic inflammation (Sendler et al., 2020). Additionally, quercetin influences immune cell polarization by promoting the anti-inflammatory M2 phenotype in macrophages and modulating the balance between pro-inflammatory Th17 cells and regulatory T cells (Tregs). These combined actions contribute significantly to reducing the inflammatory burden associated with AP, thereby preventing the development of complications such as ALI and intestinal damage (Ge et al., 2020). Thus, quercetin shows promise not only as a potential adjunctive treatment to reduce the severity of AP but also as a strategy to prevent complications commonly associated with the disease (Liu D. et al., 2022). However, while the preclinical evidence is compelling, further research is needed to investigate quercetin’s bioavailability, long-term effects, and optimal dosing protocols (Li et al., 2022). Such research will be critical in determining the clinical applicability of quercetin as a therapeutic agent for AP (Soofiyani et al., 2021; Tang et al., 2020).

This review aims to provide a comprehensive overview of the therapeutic potential of quercetin in the context of AP. By summarizing current research, we seek to elucidate the mechanisms through which quercetin modulates oxidative stress and inflammation in AP, while also discussing the challenges and opportunities in translating these findings into clinical practice. Additionally, we will identify gaps in the existing knowledge and propose future directions for research in this promising field.

## 2 Oxidative inflammatory and immune mechanisms underlying acute pancreatitis

### 2.1 Role of oxidative stress in acute pancreatitis

Oxidative stress is pivotal in the pathogenesis of AP, primarily through the induction of cellular damage and the amplification of the inflammatory response (Chen et al., 2024; Sheibani et al., 2024). The excessive generation of ROS (ROS) in AP is a critical driver of lipid peroxidation, which in turn compromises the integrity of cellular membranes and organelles (Wang et al., 2025; Li Jin-Wei et al., 2024). As lipid peroxidation escalates, it triggers cell necrosis and stimulates the release of pro-inflammatory cytokines, such as TNF-α and interleukin-6 (IL-6), thereby intensifying the inflammatory response (Li Jin-Wei et al., 2024).Furthermore, the depletion of endogenous antioxidants, including superoxide dismutase (SOD) and glutathione (GSH), exacerbates this pathological process. SOD, an essential antioxidant enzyme, facilitates the dismutation of superoxide radicals (O2−) into hydrogen peroxide (H2O2), which is subsequently decomposed by enzymes such as catalase (CAT) and GSH peroxidase, thus mitigating the deleterious effects of ROS. In the context of AP, the depletion of SOD impairs the organism’s capacity to neutralize ROS, thereby accelerating oxidative damage and exacerbating pancreatic tissue injury. Typically, these antioxidants neutralize ROS; however, their insufficient levels in AP result in ROS accumulation, thereby exacerbating oxidative damage. Cai et al. (2024) demonstrated that excessive ROS production directly causes pancreatic tissue damage and exacerbates the pathological process in animal models of AP. Their study highlights the critical role of ROS in AP, showing that ROS accumulation leads to cellular damage and amplifies the inflammatory response by activating the NF-κB pathway and inhibiting the p62-Keap1-Nrf2 pathway. Furthermore, oxidative stress induced by excessive ROS not only contributes to cellular injury but also initiates lipid peroxidation, a process that plays a key role in the inflammatory response during AP. Recent studies (Kong et al., 2021; Yang et al., 2023) have elucidated that lipid peroxidation not only compromises membrane integrity but also triggers the release of damage-associated molecular patterns (DAMPs), which serve as potent endogenous signals for immune activation. These DAMPs promote immune cell infiltration and intensify both local pancreatic and systemic inflammation. In a similar vein, Niu et al. (2024) demonstrated that Emodin confers protection against severe AP-associated lung injury by activating the Nrf2/HO-1/GPX4 signaling pathway and inhibiting ferroptosis, underscoring the pivotal role of oxidative stress in mediating not only pancreatic injury but also dysfunction in distant organs. Furthermore, Shen et al. (2025) reported that hyperlipidemia exacerbates the severity of AP through the overproduction of ROS and activation of the p38 MAPK pathway, further highlighting the pathological intersection between oxidative stress and metabolic dysregulation.

These findings collectively illustrate that oxidative stress is not merely a downstream consequence of inflammation, but rather a central pathological driver that orchestrates both local pancreatic injury and systemic complications in AP. Notably, oxidative stress and inflammation appear to be tightly interconnected, forming a self-perpetuating vicious cycle in which ROS promotes the expression of pro-inflammatory cytokines, while inflammatory mediators further enhance ROS production. This bidirectional feedback loop suggests that oxidative stress may serve as a therapeutic “hub” capable of influencing multiple downstream pathological events. Thus, oxidative stress is not just a contributing factor but a central axis in the pathophysiology of AP. Its role in driving lipid peroxidation, immune cell recruitment, and systemic inflammation makes it a compelling target for intervention. However, translating these insights into effective clinical strategies requires a nuanced understanding of the redox–inflammation interface and a shift from generalized antioxidant therapy toward more personalized, mechanism-based approaches.

### 2.2 Immune-regulatory mechanisms in acute pancreatitis

#### 2.2.1 Inflammatory pathways in acute pancreatitis

AP triggers an inflammatory response through the activation of multiple signaling pathways, primarily driven by the release of key cytokines such as TNF-α, IL-6, and IL-1β (Qiu et al., 2025; Wu et al., 2021) These cytokines initiate a cascade of inflammatory events, leading to pancreatic tissue damage and dysfunction (Sheng M. et al., 2021; Jian et al., 2024). Among the various signaling pathways involved, the NF-κB and mitogen-activated protein kinase (MAPK) pathways play particularly critical roles in amplifying inflammation and exacerbating tissue injury. The MAPK pathway, which includes ERK (Extracellular Signal-Regulated Kinase), JNK(c-Jun N-terminal Kinase), and p38 subfamilies, is a key regulator of cellular responses to stress and inflammatory stimuli. Upon activation by cytokines or oxidative stress, MAPKs modulate the transcription of pro-inflammatory genes, thereby contributing to the sustained inflammatory milieu observed in AP (Shen Y. et al., 2022). demonstrated that NF-κB serves as a pivotal transcription factor that is activated in response to cytokines or ROS. Once activated, NF-κB upregulates the expression of multiple pro-inflammatory genes, thereby intensifying immune responses and worsening pancreatic injury. This finding underscores the essential role of NF-κB in the pathophysiology of AP. In parallel, Huang et al. (2023) highlighted that the MAPK pathway, which includes p38, ERK, and JNK, contributes to inflammation by activating pro-inflammatory signaling molecules, thereby further promoting cytokine release and cellular damage. Collectively, these studies emphasize the central roles of the NF-κB and MAPK pathways in the inflammatory processes of AP and suggest that targeting these pathways may represent an effective therapeutic approach. Building on this understanding, additional research has explored potential therapeutic interventions aimed at suppressing these pathwaysIn summary, these studies not only elucidate the complexity of inflammatory signaling pathways in AP but also underscore the critical importance of targeting multiple pathways for effective therapeutic intervention. Notably, quercetin’s ability to modulate key inflammatory pathways further reinforces its potential as a promising therapeutic agent for AP.

#### 2.2.2 Macrophage polarization and pyroptosis in acute pancreatitis

Macrophages are classified into two main types based on their polarization: pro-inflammatory M1 macrophages and anti-inflammatory M2 macrophages (Wang and Mei, 2023; KhaterCA et al., 2024). M1 macrophages are key drivers of inflammation, characterized by the release of cytokines such as TNF-α and IL-6, which amplify the inflammatory cascade and contribute to tissue damage. In contrast, M2 macrophages secrete anti-inflammatory cytokines, including IL-10, and play a crucial role in tissue repair and the resolution of inflammation (He et al., 2025; Sahana et al., 2022). The dynamic balance between M1 and M2 macrophages is critical in determining the extent of inflammation in AP. Ryu et al. (Liu et al., 2024) examined the role of macrophages in pancreatic diseases, with a focus on how macrophage polarization impacts these conditions. Their research demonstrated that macrophages are central to the pathogenesis of pancreatic diseases, with M1 macrophage polarization being linked to exacerbated inflammation in pancreatitis. Moreover, Liu et al. (2024) used *ELAV1* gene knockout mice to develop an AP model, revealing that *ELAV1* upregulates TRAF6 expression, which enhances TLR signaling and promotes M1 macrophage polarization, thereby worsening the course of AP. In addition to macrophage polarization, macrophage pyroptosis also plays a pivotal role in AP. Bansod et al. (2020), and Peng et al. (2024) showed that the cGAS-STING pathway, through activation of IRF7/IRF3, promotes pyroptosis in macrophages, which aggravates lung injury caused by AP. This finding underscores the important role of macrophage pyroptosis in amplifying the inflammatory response. Furthermore, Sheng Bo et al. (2021) investigated the impact of exosomal miR-181a-5p derived from bone marrow-derived mesenchymal stem cells (BMSCs) on macrophage polarization. They found that upregulation of miR-181a-5p promoted M2 macrophage polarization, leading to a significant reduction in pancreatic damage and inflammation, thereby suggesting that M2 macrophages play a crucial protective role in resolving inflammation. In conclusion, macrophage polarization and pyroptosis play critical roles in the progression of AP. Specifically, the heightened polarization of M1 macrophages and the potential protective effects of M2 macrophages in inflammation resolution and tissue repair underscore the importance of modulating these processes. Targeting macrophage polarization and pyroptosis presents promising therapeutic opportunities for AP and may contribute to the development of more targeted, immune-based treatments.

#### 2.2.3 T Cell subset alterations in acute pancreatitis

In AP, T cell dysregulation often exacerbates disease progression, with Th17 cells playing a pivotal role in this process. Boodhoo et al. (2022) examined the involvement of T cells in AP and identified Th17 cells as critical mediators in its pathogenesis. Through the secretion of pro-inflammatory cytokines su as IL-17, Th17 cells promote the release of additional inflammatory mediators, thereby contributing to pancreatic tissue damage. This suggests tha excessive activation of Th17 cells significantly amplifies both inflammation and tissue injury in AP. Additionally, a reduction in Treg cell funcion is recognized as a key factor driving immune dysregulation in AP. The depletion of Treg cells results in impaired immune regulation, exacerating the inflammatory response and further aggravating tissue damage. Lethen et al. (2024) demonstrated that the Th17/Treg imbalance i AP patients correlates with disease severity, indicating that dysregulation of these T cell subsets may be a critical determinant of poor clinicaoutcomes. Beyond alterations in T cell subsets, T cell receptor (TCR) rearrangement also plays an integral role in the immune response during A. Wang et al. (Ryu and Lee, 2024) highlighted that changes in the TCR repertoire of peripheral blood T cells are significantly associated witthe progression of pancreatic disease, suggesting that TCR repertoire alterations are an inherent part of the immune response in AP. In conclusin, the functional perturbations of T cells, particularly the Th17/Treg imbalance, are central to the initiation and perpetuation of inflammatio in AP. Modulating T cell subset ratios and restoring the Th17/Treg balance may offer a promising therapeutic strategy for AP. Furthermore, alteratons in the TCR repertoire could serve as novel biomarkers for immune monitoring and disease prognosis. Thus, a comprehensive understandig of the mechanisms underlying T cell changes in AP is essential for the development of innovative immune-targeted therapies.

## 3 Therapeutic potential of quercetin in acute pancreatitis

### 3.1 Qurcetin’s role in attenuating oxidative stress

**FIGURE 2:**
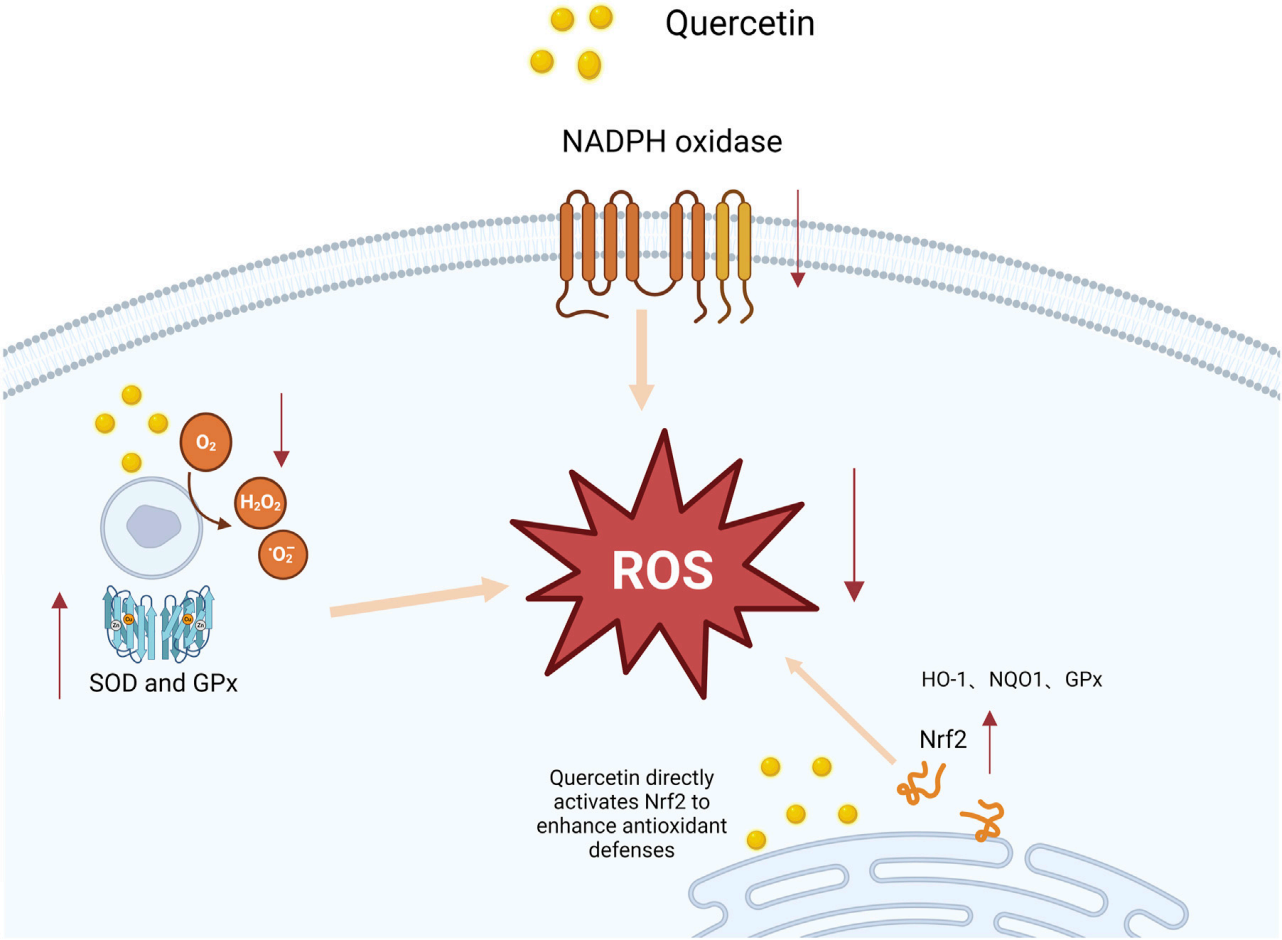
Mechanism of quercetin-induced Nrf2 activation and enhanced antioxidant defenses. This figure illustrates how quercetin interacts with nicotinamide adenine dinucleotide phosphate (NADPH) oxidase, resulting in an increase in reactive oxygen species (ROS). The elevated ROS levels activate the nuclear factor erythroid 2-related factor 2 (Nrf2) pathway, which subsequently promotes the expression of antioxidant genes, such as superoxide dismutase (SOD) and glutathione peroxidase (GPx). As a result, the antioxidant response is potentiated, reducing oxidative stress and protecting cells from damage.

Quercein, a natural flavonoid compound, exhibits significant antioxidant properties, effectively inhibiting ROS and lipid peroxidation, while promotingthe synthesis of intracellular GSH (GSH). Zhou et al. (2025); Hossein et al. (2024) observed in their study that quercetin significanty inhibits ROS production, demonstrating preventive effects on endothelial dysfunction in chronic kidney disease. Hossein et al. (2024) urther confirmed in cell experiments that quercetin treatment of oxidative stress-induced endothelial cells significantly reduced ROS levels, increased SH levels, and decreased cell apoptosis, highlighting quercetin’s prominent role in antioxidant defense. Additionally, several studies suggest tht quercetin can directly scavenge free radicals and enhance the activity of antioxidant enzymes in pancreatic tissues, thereby significantly reducing oidative stress. Faiza et al. (2024) found that in a cerulein-induced AP model, quercetin significantly reduced ROS productionand enhanced antioxidant enzyme activities, including SOD, CAT, and GSH peroxidase (GSH-Px). These findings further confirm quercetin’ potential in improving oxidative stress balance. Not only does quercetin reduce oxidative damage by scavenging free radicals, but it also upregulate the expression of endogenous antioxidant enzymes, helping restore oxidative balance in damaged pancreatic cells. Qi et al. (2022) repoted that quercetin upregulates the Nrf2 pathway, enhancing the cell’s antioxidant defense mechanisms. The Nrf2 pathway is crucial in the cellular resonse to oxidative stress, and by promoting Nrf2 activation, quercetin increases GSH synthesis, thereby improving the redox balance in pancreatic issues. This mechanism effectively mitigates oxidative damage in AP, further supporting the potential of quercetin as an antioxidant therapy, asdepicted in Figure 2. In conclusion, quercetin exhibits multifaceted potential in reducing oxidative stress by scavenging free radical pathways, and activating the Nrf2 pathway. These findings confirm its protective effect on pancreatic tissues and highlight its significance in systemic pathological responses, underscoring its promising therapeutic potential and providing a solid foundation for clinical application.

### 3.2 The immunoregulatory effects of quercetin

#### 3.2.1 Quercetin’s suppression of inflammatory signaling

**FIGURE 3:**
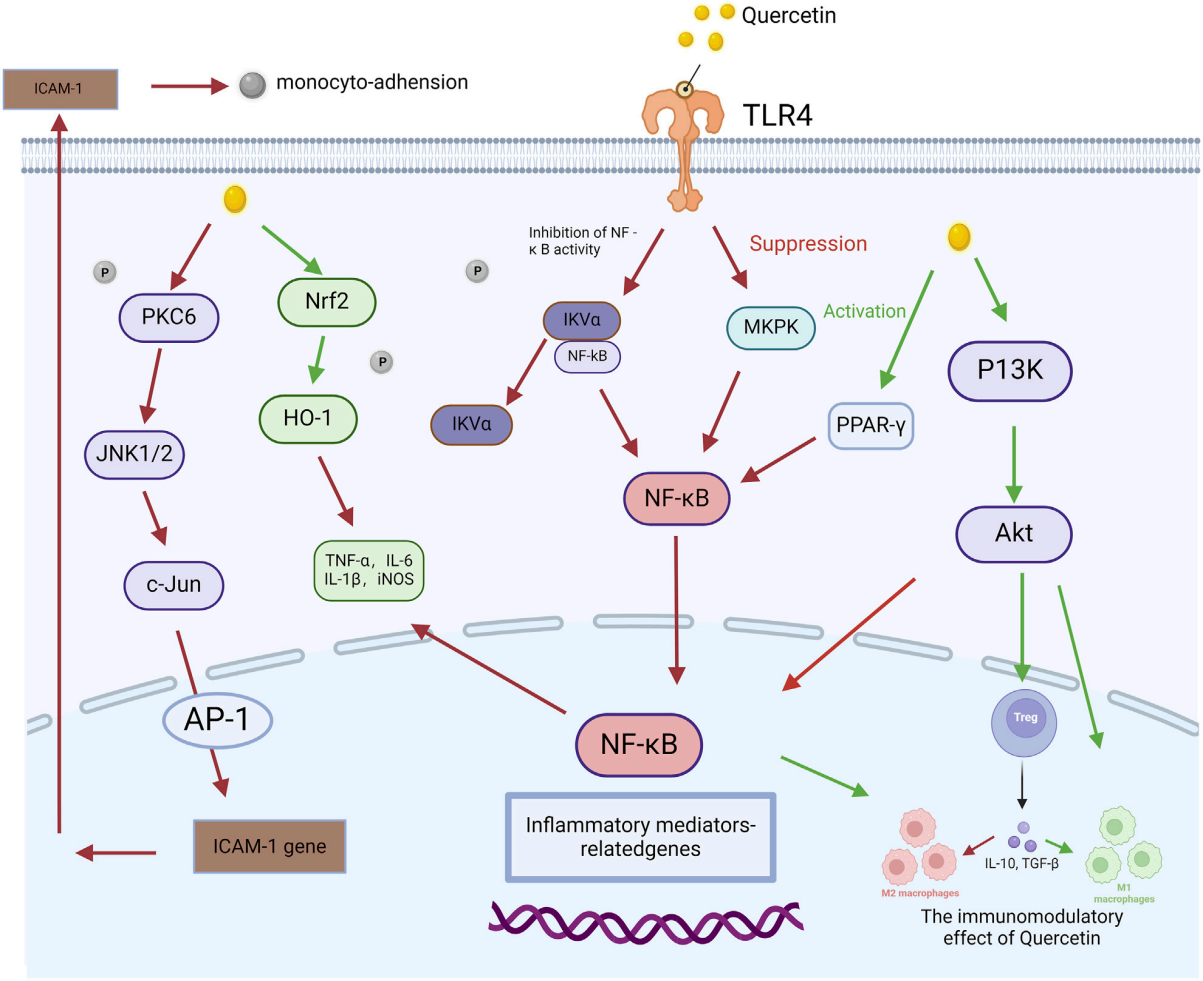
Quercetin-mediated signaling pathway in the regulation of acute pancreatitis. This figure illustrates how quercetin modulates the molecular pathways involved in AP. It begins by activating Toll-like receptor 4 (TLR4), initiating a cascade of signaling events involving molecules such as protein kinase C delta (PKCδ), c-Jun N-terminal kinase 1/2 (JNK1/2), and c-Jun. These molecules subsequently interact with nuclear factor erythroid 2-related factor 2 (Nrf2) and heme oxygenase-1 (HO-1), influencing the production of pro-inflammatory cytokines, including tumor necrosis factor-alpha (TNF-α), interleukin-6 (IL-6), interleukin-1 beta (IL-1β), and inducible nitric oxide synthase (iNOS). Quercetin also attenuates nuclear factor-kappa B (NF-κB) activation by inhibiting inhibitor of kappa B-alpha (IκBα), leading to reduced expression of inflammation-related genes. Additionally, quercetin activates peroxisome proliferator-activated receptor gamma (PPAR-γ), which mediates anti-inflammatory effects. This figure highlights the key molecular processes through which quercetin mitigates inflammation in AP.

Quercetin has been extensively studied for its anti-inflammatory properties, particularly in the context of AP, where it alleviates the inflammatory response by inhibiting the NF-κB and MAPK signaling pathways. For example, Rezabakhsh et al. (2019) found that quercetin significantly reduced pancreatic edema and necrosis in animal models of pancreatitis by inhibiting the activation of both NF-κB and TLR4 pathways. Similarly, Liu Y. et al. (2022) demonstrated that quercetin significantly suppressed the activation of the NF-κB and MAPK pathways in AP by modulating miR-216b, targeting *MAP2K6* and *NEAT1*, thereby further highlighting its potential therapeutic role in mitigating this condition. These findings are further corroborated by subsequent research, which continues to support quercetin’s efficacy in managing inflammation in AP. For example, Li H. et al. (2024) reported that quercetin regulates the TLR-mediated MAPK and Akt signaling pathways in hypercholesterolemic rats, leading to a significant reduction in inflammation. This result extends the anti-inflammatory effects of quercetin, suggesting its potential efficacy not only in pancreatitis but also in other inflammatory diseases involving these pathways. Additionally, Wang et al. (2022) further confirmed that quercetin alleviates kidney injury in pregnant mice with acute necrotizing pancreatitis by inhibiting the p38 MAPK pathway. The discovery highlights the crucial role of the MAPK pathway in driving inflammation and demonstrates that quercetin effectively mitigates pancreatic and renal damage by targeting this mechanism.These findings demonstrate the substantial therapeutic potential of quercetin in mitigating inflammatory responses through multi-pathway inhibition. A visual overview of the key inflammatory signaling pathways, such as NF-κB and MAPK, which are modulated by quercetin, is provided in Figure 3. Collectively, these studies support the clinical application of quercetin as a multi-target therapeutic agent, indicating its potential to mitigate AP and related inflammatory damage through the inhibition of the NF-κB and MAPK signaling pathways.

#### 3.2.2 Modulation of macrophage polarization by quercetin

Quercetin has been shown to effectively modulate macrophage polarization by shifting the balance from the M1 to the M2 phenotype, thereby fostering an anti-inflammatory microenvironment. This modulation is achieved through its regulation of inflammatory signaling pathways and its ability to enhance the expression of M2 macrophage markers, such as CD206 and arginase-1, while simultaneously suppressing M1-associated cytokines, including TNF-α and IL-6 (Wang et al., 2022); Gao et al., 2024. For instance, (Guo et al., 2020) demonstrated that quercetin treatment significantly reduced the population of M1 macrophages and enhanced M2 macrophage activity in a cerulein-induced AP model, resulting in reduced tissue inflammation and accelerated healing. Additionally, recent studies have highlighted quercetin’sability to target key molecular pathways involved in macrophage polarization. Wang et al. (2021) reported that quercetin, as a major component of Xiang-lian Pill, activates the peroxisome proliferator-activated receptor gamma (PPAR-γ) pathway, tipping the balance of STAT1/PPARγ, and promoting the alternative activation of macrophages to favor M2 polarization. This study highlights quercetin’s role in modulating macrophage activity and reducing inflammation in related inflammatory conditions. Similarly, Sheng B. et al. (2021) found that quercetin inhibits NF-κB activation, reducing the pro-inflammatory cytokine production associated with M1 macrophages while promoting an anti-inflammatory milieu. By enhancing the M2 phenotype and reducing M1-driven inflammation, quercetin creates a favorable environment for resolving inflammation and facilitating tissue repair. These findings strongly support the therapeutic potential of quercetin as an immunomodulatory agent in AP. Future research should explore the translational implications of quercetin in clinical settings, particularly its integration into multi-target therapies for managing inflammatory and immune dysregulation in AP.

#### 3.2.3 Modulation of T Cell subset balance in immune responses by quercetin

Currently, there is no direct evidence in the literature indicating that quercetin can treat AP by modulating the balance of T cell subsets (Bhaskar and Helen, 2016; Zhang et al., 2021). However, existing studies suggest that the anti-inflammatory mechanisms of quercetin are closely linked to the regulation of T cell subsets (Zhou et al., 2023; Lin et al., 2023). In a study by Hai-feng et al. (2023) and Yang et al. (2018) investigating collagen-induced arthritis, it was found that quercetin exerts its anti-inflammatory effects by restoring the balance between Th17 and Treg cells, thereby promoting an increase in Treg cell proportion. Furthermore, Chen et al. (2021) and Wei et al. (2017) emphasized that quercetin regulates the Th17/Treg balance through the Tim-3 and TLR4-MyD88-NF-κB signaling pathways, thus contributing to its anti-inflammatory and hepatoprotective effects. Given that advanced stages of AP are often associated with a heightened risk of progression to pancreatic cancer, some studies (Shen M. et al., 2022)suggest that quercetin may also influence the JAK/STAT1 signaling pathway, promoting the proliferation of γδ T cells. This modulation plays a synergistic role in the elimination of breast cancer cells and the induction of cell apoptosis. Additionally, quercetin enhances the immune response, reducing the evasion of tumor cells from immune surveillance. (Shen M. et al., 2022) demonstrated, through a co-culture system of breast cancer cells and T cells treated with quercetin, that quercetin significantly increased the proportion of γδ T cells, enhancing T cell immune responses, and inhibiting both the proliferation and migration of breast cancer cells. These findings underscore quercetin’s significant immune-modulatory effects, particularly its potential to regulate the balance between Th17 and Treg cells (Zhou et al., 2024); Yu et al., 2024. In the context of AP, such mechanisms may help mitigate inflammation and improve disease outcomes by restoring the balance between pro-inflammatory Th17 cells and anti-inflammatory Treg cells. Further investigation into quercetin’s role in modulating immune responses in AP could provide novel therapeutic strategies for managing inflammation in this condition.

### 3.3 Advantages and challenges of quercetin in clinical applications

#### 3.3.1 Comparison of quercetin with conventional therapies for acute pancreatitis

Conventional therapies for AP, such as N-acetylcysteine (NAC) and corticosteroids, primarily target specific disease components but fail to offer a comprehensive solution for the multifactorial pathogenesis of the disease (Qiu et al., 2021). NAC alleviates oxidative stress by replenishing GSH levels and neutralizing ROS. However, NAC’s efficacy is limited in its inability to address the inflammatory response and immune dysregulation, which are critical in the progression of AP (Fang et al., 2024; Minati et al., 2021). For example, a study by Ma et al. (2019) on NAC in patients with non-alcoholic fatty liver disease (NAFLD) showed that while NAC helped reduce oxidative stress markers, it failed to significantly improve inflammatory cytokine levels or liver function in the long term, highlighting NAC’s gap in managing inflammation. This finding highlights a critical consideration regarding the limited therapeutic scope of NAC, as it may alleviate oxidative damage but does not comprehensively address the immune inflammatory cascade responsible for systemic complications in AP. This emphasizes the necessity for the development of therapeutic strategies that can concurrently target oxidative stress and inflammation in a synergistic manner.Corticosteroids, on the other hand, are widely used as potent anti-inflammatory agents in treating AP and other inflammatory conditions. However, their long-term use is restricted due to a range of adverse effects, such as immune suppression, increased infection risk, and metabolic disturbances like hyperglycemia.For instance, in rheumatoid arthritis (RA) patients, long-term corticosteroid use has been associated with increased rates of infections and osteoporosis, as well as delayed wound healing, which severely hampers recovery (Rifaai et al., 2012). A critical issue with corticosteroids in the context of AP is their narrow therapeutic window, as they reduce inflammation but their immune-suppressive effects may actually hinder tissue repair and host defense, particularly in a setting where infection risk is high. This paradoxical outcome challenges the assumption that corticosteroids can be universally effective in inflammatory diseases, urging caution in their clinical application.

In contrast, quercetin, a flavonoid with potent antioxidant and anti-inflammatory properties, offers a broader therapeutic approach by simultaneously addressing oxidative stress and inflammation. Quercetin enhances the activity of endogenous antioxidants like SOD and GSH-Px, providing robust protection against oxidative damage and preserving cellular integrity (Huang et al., 2023; Sahana et al., 2022). For instance, Minati et al. (2021) demonstrated that quercetin offers protective effects on pancreatic tissue by attenuating inflammatory responses and preserving immune homeostasis, as observed in experimental diabetes models.In human clinical trials for other diseases, quercetin has demonstrated remarkable potential. For example, a study by Zhu et al. (2024) on quercetin supplementation in patients with type 2 diabetes found that quercetin not only reduced oxidative stress but also significantly decreased inflammatory markers like C-reactive protein (CRP) and TNF-α, while improving insulin sensitivity. This suggests that quercetin’s multi-targeted action may offer more comprehensive benefits compared to NAC and corticosteroids, especially in diseases where both oxidative stress and inflammation contribute to the pathological process. However, while these findings are promising, the lack of large-scale clinical trials specifically investigating quercetin in AP remains a gap in the literature, and its therapeutic potential must be tested in rigorous clinical settings.Additionally, quercetin’s immunomodulatory effects, particularly its ability to promote M2 macrophage polarization, enhance the resolution of inflammation and facilitate tissue repair—key processes in the recovery from AP. In preclinical studies, quercetin has been shown to promote M2 macrophage polarization, which plays a critical role in tissue repair and the resolution of inflammation. For instance, in experimental colitis, quercetin has been shown to shift macrophages from the pro-inflammatory M1 phenotype to the anti-inflammatory M2 phenotype, thereby aiding in tissue regeneration and reducing inflammation (Oliveira et al., 2019). This contrasts with the inflammatory exacerbation typically observed with prolonged use of corticosteroids, which can impair immune cell function and delay healing. Quercetin’s multi-targeted action thus offers distinct advantages over traditional therapies by simultaneously addressing oxidative stress, inflammation, and immune modulation, while minimizing side effects As shown in Table 1.

**TABLE 1:** Comparison of quercetin with conventional therapies for acute pancreatitis.

Comparison Criteria	Quercetin	Conventional therapies (e.g., N-Acetylcysteine, corticosteroids)	References
Mechanism of Action	Multi-target approach: Antioxidant, anti-inflammatory, and immunomodulatory effects. Reduces oxidative stress and inflammation	NAC: Primarily replenishes glutathione levels to combat oxidative stress; Corticosteroids: Primarily anti-inflammatory (NF-κB suppression)	Li et al., 2022; Ma et al., 2019
Oxidative Stress Reduction	Increases the activity of endogenous antioxidants (e.g., SOD, catalase). Enhances mitochondrial function and reduces ROS production	NAC: Enhances glutathione levels but does not directly impact inflammation or immune modulation	Qiu et al. (2025), Zhu et al. (2024), Raghu et al. (2021)
Inflammation Modulation	Inhibits key inflammatory pathways (NF-κB, MAPK), reduces pro-inflammatory cytokines (TNF-α, IL-6, IL-1β), promotes M2 macrophage polarization	Corticosteroids reduce inflammation but risk immune suppression and metabolic side effects	Ma et al. (2019), Rifaai et al. (2012), Tanomrat et al. (2023)
Immunomodulation	Promotes the polarization of macrophages from M1 (pro-inflammatory) to M2 (anti-inflammatory), aiding in tissue repair	Corticosteroids can suppress immune function, increasing the risk of infections	Lethen et al. (2024), Martínez et al. (2019)
Bioavailability	Poor bioavailability; requires nanoparticle or liposomal formulations for enhanced absorption and efficacy	NAC has good oral bioavailability, but corticosteroids can cause long-term side effects like metabolic disturbances	Fang et al. (2024)
Side Effects	Few documented side effects in preclinical studies; further human trials needed to confirm long-term safety	Corticosteroids may cause immune suppression, osteoporosis, and adrenal insufficiency with long-term use	Oliveira et al. (2019)
Clinical Efficacy	Promising results in animal studies but limited clinical trials. Shows potential as an adjunct therapy	NAC and corticosteroids are commonly used but do not address all aspects of the disease, especially immune modulation	Zhu et al. (2024)

While quercetin’s ability to target both biochemical pathways and immune responses positions it as a potentially safer and more comprehensive alternative to conventional treatments, it is important to note that current evidence largely stems from preclinical studies and smaller clinical trials. The full clinical validation of quercetin’s efficacy in treating AP is still needed. Emerging clinical data also suggest that quercetin may work synergistically with existing therapies, enhancing their therapeutic outcomes without exacerbating side effects. In this sense, quercetin represents not only a potential adjunct but also a novel therapeutic avenue, warranting further investigation in clinical trials.

#### 3.3.2 The limitations of quercetin in the treatment of pancreatitis

Although quercetin demonstrates significant therapeutic potential in managing AP, its clinical application is hindered by several challenges. A primary limitation is its poor bioavailability, which arises from its low solubility and rapid metabolism in the liver (Barbulescu et al., 2023). This limitation restricts its therapeutic efficacy, particularly in severe inflammatory conditions like AP. While pharmacokinetic modifications such as nanoparticles, liposomal carriers, and co-administration with bioenhancers like piperine have shown promise in enhancing quercetin’s absorption, tissue targeting, and circulation time, the long-term clinical validation of these strategies remains limited (Ansari et al., 2022). Preclinical studies suggest that these delivery methods could optimize quercetin’s bioavailability, but human studies are sparse and often limited to small sample sizes. For instance, while a study by Kumar et al. (2022) on liposomal quercetin demonstrated improved bioavailability in cancer models, its translation into diseases like AP, with its complex pathophysiology and high systemic inflammatory load, requires more robust clinical testing.Further complicating matters, quercetin’s bioavailability issue is often addressed with adjunct therapies, such as piperine, which enhances quercetin absorption by inhibiting liver enzymes involved in its metabolism. However, the safety profile of this combination, particularly in patients with pre-existing liver conditions or drug interactions, remains underexplored. A critical question arises: while bioenhancers may improve quercetin’s efficacy, do they also increase the risk of toxicity, especially when quercetin is co-administered with other potent drugs like corticosteroids or NAC? This concern underscores the need for careful dose adjustment and patient monitoring during quercetin-based therapies.

Moreover, despite promising preclinical data, the lack of large-scale clinical trials hampers our ability to establish optimal dosages, long-term safety, and the full range of potential side effects. While quercetin has demonstrated anti-inflammatory and antioxidant effects in animal models and *in vitro* studies, its therapeutic application in human pancreatitis remains speculative. For instance, studies on quercetin’s use in type 2 diabetes and cardiovascular diseases suggest efficacy in reducing oxidative stress and inflammatory markers (Kumar et al., 2022); however, these findings are not easily transferable to the highly complex inflammatory environment of AP. This raises a pivotal issue: while quercetin’s potential as a synergistic agent in combination with therapies like NAC or corticosteroids holds promise, its clinical application in pancreatitis is still in its infancy. It is crucial to conduct larger randomized controlled trials to evaluate quercetin’s long-term benefits and risks in the context of AP, particularly in high-risk populations such as those with comorbid conditions.

To enhance treatment outcomes, combination therapies that pair quercetin with agents like NAC or corticosteroids may effectively address both oxidative stress and inflammation, improving therapeutic efficacy while minimizing adverse effects (Eity et al., 2024). However, caution is warranted when using such combinations, as NAC itself has limitations in treating the immune-inflammatory response, and corticosteroids carry the risk of immunosuppression. More research is needed to determine whether quercetin’s ability to modulate immune pathways can counterbalance these potential drawbacks in combination therapies.

## 4 Future directions

Quercetin holds significant promise as a multi-target therapeutic in AP, thanks to its antioxidant, anti-inflammatory, and immune-modulatory properties (Sun et al., 2025). However, its clinical application is limited by poor bioavailability, which affects its efficacy, particularly in severe conditions like AP. Nanoparticles and liposomal formulations have shown potential to improve its bioavailability, but these approaches need extensive validation to assess long-term safety and clinical relevance. While preclinical models show positive results, there is a critical gap in data regarding optimal dosing, side effects, and drug interactions with agents like NAC and corticosteroids (Dhanya, 2022); Xing et al., 2024. This warrants caution, as combining quercetin with these therapies could either enhance or undermine their therapeutic effects.Beyond AP, quercetin’s fibrosis-reducing and tissue-healing effects suggest that it may also hold promise for treating chronic pancreatitis and other pancreatic disorders (Georgiou et al., 2023). However, the lack of robust clinical trials in chronic conditions and comorbid patients highlights the need for more targeted research. Importantly, quercetin’s dual action on inflammation and fibrosis may offer advantages over existing treatments but also raises concerns about the balance between its anti-inflammatory and potential immunosuppressive effects.Future studies should focus not only on optimizing formulations but also on exploring quercetin’s long-term safety in real-world conditions, especially in combination therapies. Addressing these clinical gaps will be key to unlocking quercetin’s full potential as a therapeutic agent for acute and chronic pancreatic diseases.

In summary, quercetin’s ability to target multiple pathological mechanisms, including oxidative stress, inflammation, and immune dysregulation, makes it a promising candidate for the treatment of AP. Its unique multi-target profile offers distinct advantages over single-target therapies. Although current preclinical data are promising, further clinical validation is essential to establish its efficacy and safety. With continued research and clinical trials, quercetin has the potential to transform the management of pancreatitis and related inflammatory disorders, advancing from the preclinical stage to widespread therapeutic use.

## Abbreviations

AP, Acute Pancreatitis; ALI, Acute Lung Injury; ARDS, Acute Respiratory Distress Syndrome; BMSC, Bone marrow-derived mesenchymal stem cell; SIRS, Systemic Inflammatory Response Syndrome; MODS, Multi-Organ Dysfunction Syndrome; ROS, Reactive Oxygen Species; NF-κB, Nuclear Factor-kappa B; MAPK, Mitogen-Activated Protein Kinase; TNF-α, Tumor Necrosis Factor-alpha; IL-6, Interleukin-6; IL-1β, Interleukin-1 beta; SOD, Superoxide Dismutase; CAT, Catalase; GSH-Px, Glutathione Peroxidase; GSH, Glutathione; TLR, Toll-Like Receptor; PPAR-γ, Peroxisome Proliferator-Activated Receptor Gamma; STAT1, Signal Transducer and Activator of Transcription 1; Nrf2, Nuclear Factor Erythroid 2-Related Factor 2; NAC, N-Acetylcysteine; HMGB1, High Mobility Group Box 1; ERK, Extracellular Signal-Regulated Kinase; JNK, c-Jun N-terminal Kinase; DAMPs, Damage-Associated Molecular Patterns.
